# Online compensation detecting for real-time reduction of compensatory motions during reaching: a pilot study with stroke survivors

**DOI:** 10.1186/s12984-020-00687-1

**Published:** 2020-04-28

**Authors:** Siqi Cai, Xuyang Wei, Enze Su, Weifeng Wu, Haiqing Zheng, Longhan Xie

**Affiliations:** 1 Shien-Ming Wu School of Intelligent Engineering, South China University of Technology, Guangzhou, 510640 China; 2 The Third Affiliated Hospital, Sun Yat-sen University, Guangzhou, 510630 China

**Keywords:** Stroke, Compensation, Machine learning, Virtual reality, Rehabilitation robot

## Abstract

**Background:**

Compensations are commonly observed in patients with stroke when they engage in reaching without supervision; these behaviors may be detrimental to long-term functional improvement. Automatic detection and reduction of compensation cab help patients perform tasks correctly and promote better upper extremity recovery.

**Objective:**

Our first objective is to verify the feasibility of detecting compensation online using machine learning methods and pressure distribution data. Second objective was to investigate whether compensations of stroke survivors can be reduced by audiovisual or force feedback. The third objective was to compare the effectiveness of audiovisual and force feedback in reducing compensation.

**Methods:**

Eight patients with stroke performed reaching tasks while pressure distribution data were recorded. Both the offline and online recognition accuracy were investigated to assess the feasibility of applying a support vector machine (SVM) based compensation detection system. During reduction of compensation, audiovisual feedback was delivered using virtual reality technology, and force feedback was delivered through a rehabilitation robot.

**Results:**

Good classification performance was obtained in online compensation recognition, with an average F1-score of over 0.95. Based on accurate online detection, real-time feedback significantly decreased compensations of patients with stroke in comparison with no-feedback condition (*p* < 0.001). Meanwhile, the difference between audiovisual and force feedback was also significant (*p* < 0.001) and force feedback was more effective in reducing compensation in patients with stroke.

**Conclusions:**

Accurate online recognition validated the feasibility of monitoring compensations using machine learning algorithms and pressure distribution data. Reliable online detection also paved the way for reducing compensations by providing feedback to patients with stroke. Our findings suggested that real-time feedback could be an effective approach to reducing compensatory patterns and force feedback demonstrated a more enviable potential compared with audiovisual feedback.

## Background

Stroke is the leading cause of long-term disability in adults worldwide [1], and many poststroke patients suffer from varying degrees of upper extremity motor dysfunction [2]. Skilled reaching is an important aspect of upper limb motor ability but is impaired after stroke [3, 4]. Patients with stroke usually develop adaptive compensatory patterns, particularly by recruiting excessive trunk or shoulder movements during reaching [5, 6]. The use of compensatory motions could be beneficial for an immediate improvement in function; however, such a functional improvementoccurs because of a reinforcement of compensation instead of true motor recovery [7]. Patients with stroke who commonly use compensatory strategies may form nonoptimal motion patterns, hindering long-term recovery of their impaired arms [8, 9]. Previous studies have demonstrated that reducing compensatory patterns has the potential to improve the final functional outcome. Improvements were accompanied by lager active joint range [8], higher FMA-UE score [10] and recovery of lost motor patterns [7]. Therefore, therapists correct undesired compensatory motions when they supervise therapeutic exercises. However, stroke patients perform many exercises without supervision, such as home therapy, which highlights the need to detect and reduce compensationin unsupervised rehabilitation [10].

Automatic detection of compensation can ensure subsequent intervention to prompt the patient into the correct pose. Previous studies have evaluated the feasibility of sensor-based and camera-based systems to detect compensation without the supervision of a therapist [11–15]. However, camera systems are not appropriate for application in clinical or home settings, which face challenges such as object obstruction, complex setups and privacy [13, 16]. Sensor-based systems suffer from inducing unnatural motions due to the attachment of sensors. Moreover, the reliability of the outcome estimates from these sensors is still a challenge for researchers [14, 16]. While the detection of compensatory patterns still lacks a simple, unobtrusive and practical method, we have proposed a pressure distribution-based compensation detection system [17, 18]. With a pressure mattress mounted on the chair, participants performed seated reaching tasks, and the pressure distribution data were recorded. Several features were extracted from the pressure distribution data that reflected the information for different kinds of compensatory motions. Different models were applied to recognize compensatory patterns and achieved excellent offline recognition accuracy. Our previous studies pave the path toward detecting compensation based on pressure distribution data and machine learning methods. However, there is still a gap between online and offline detection performance, and few studies on the real-time detection of compensation have been reported. To our knowledge, no previous study has evaluated the feasibility and validity of detecting compensatory motions based on pressure distribution data and machine learning methods in real time. Therefore, the purpose of this study is to investigate whetherthe pressure distribution-based method can be implemented in the real-time monitoring of compensatory motions in patients with stroke.

Based on the real-time detection of compensation, various feedback strategies, in the form of visual [19, 20], auditory [21, 22], or force feedback [23], were provided to patients with stroke to modify their motion patterns. However, there is still no consensus on the kind of feedback modalities that would be effective in reducing compensation. In this study, virtual reality (VR) technology was employed to provide audiovisual feedback, while a rehabilitation robot was employed to provide force feedback. This pilot study aimed to investigate whether the compensation of stroke survivors during reaching can be reduced by audiovisual and force feedback and to examine whether one feedback method is superior to the other.

Therefore,the main contributions of this paper are as follows:

1. The implementation and validation of thepresented compensation-detecting method using pressure distribution data and machine learning algorithms in real time;
2. The use of virtual reality and a rehabilitation robot to reduce compensatory motions in patients with strokeduring reaching tasks; and
3. The comparison of audiovisual and force feedback for reducing compensation.

## Methods

### Participants

Eight poststroke subjects were recruited from the Third Affiliated Hospital, SUN Yat-sen University. A summary of the participants’ demographics was provided in Table 1. Ethical approval was obtained from the Guangzhou First People’s Hospital Department of Ethics Committee. All participants reviewed and signed an informed consent form prior to entering the study.

**Table 1:** Demographic and clinical data for participants with stroke

Subject	Age	Gender	Height (cm)	Weight (kg)	Affectedside	Months post stroke	MMSE	FMA
S1	54	F	150	60	Left	2	26	34
S2	45	M	164	54	Left	3	30	55
S3	68	F	155	39.5	Left	2	30	38
S4	52	M	175	65	Left	6	27	19
S5	37	M	173	72.5	Right	5	30	32
S6	65	M	172	65	Right	9	26	35
S7	65	M	170	51	Left	4	25	36
S8	66	M	167	65	Left	3	27	29
Average ± SD	56.5 ± 10.64		165.8 ± 8.39	59 ± 9.75		4 ± 2	27.6 ± 1.93	35 ± 9

Participants were included if they had experienced their first-ever stroke, if they were either in the subacute (between 1 to 6 months poststroke) or chronic (over 6 months poststroke) stage of recovery, if they were able to remain in a sitting posture (without back or arm rests) for at least 10 min and if they were able to perform the required motions. Participants were excluded from the study if they had the following: upper limb pain > 4/10 on a visual analogue scale (VAS) [24]; upper limb spasticity > 2 on the Modified Ashworth Scale (MAS) [25]; Mini-Mental State Examination (MMSE) score ≤ 23 [26]; or visual spatial neglect based on clinical judgment.

### Experimental setup

**Fig. 1:**
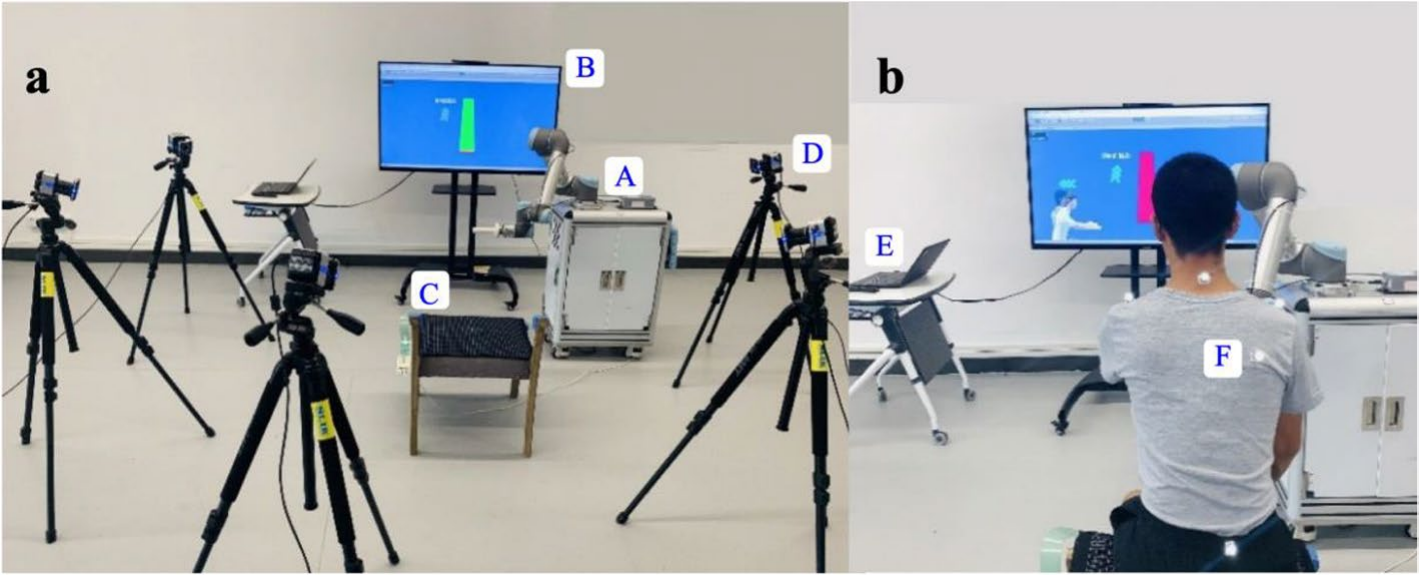
The experimental setup. (**a**) and (**b**) display the integrated platform, including (A) The rehabilitation robot, (B) TV screen (display deviceof virtual reality), (C) pressure distribution mattress, (D) 3D motion capture system, (E) computer and (F) reflective markers

The integrated platform comprised a pressure distribution mattress (Body Pressure Measurement System (BPMS), Model 5330, Tekscan, Inc., South Boston, MA, USA), a rehabilitation robot, 3D motion capture system (VICON, Oxford Metrics, UK), and a personal computer (see details in Fig. 1). The BPMS system was mounted on the chair to record the pressure distribution data of each patient during the seated reaching tasks. The rehabilitation robot, which is based on UR5 (Universal Robots Ltd., Odense, Denmark) [27], can support and guide the movement of the impaired arm in 3D space. A handle was attached at the end of UR5 for participants to hold. An admittance control scheme [28–30], which is a position controller with force feedback, was implemented. A 6-DOF force sensor is attached to the end effector of the ReRobot, then the input from this sensor is used to produce velocity commands for the device. The rehabilitation robot can be commanded to maintain the correct direction or provide haptic feedback in the form of assistive force to the subject. And the assistive force was provided based on the ‘assistance-as-needed’ principle [31, 32], that is, the ReRobot can adaptively generate the necessary assistive force based on the estimated user’s force output. Transmission Control Protocol/Internet Protocol (TCP/IP) was used for communication between the rehabilitation robot and the MATLAB (MathWorks Corp., Natick, MA, USA) user interface. The virtual environments (VEs) were employed to provideaudiovisual feedback, which was built in Unity3D (Unity Technologies, CA, USA) [33] and connected to the rehabilitation robot and pressure distribution mattress through the TCP/IP. The pressure distribution data were recorded at 50 Hz. The VICON system was used to track participants’ upper limb and trunk movements at 100 Hz.

**Fig. 2:**
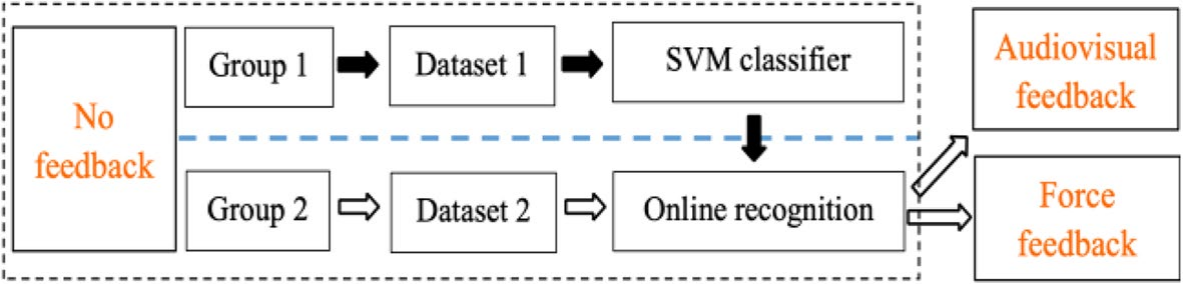
Experimental design

The experiment consisted of three sessions, including no feedback,audiovisual feedback and force feedback,as shown in Fig. 2. Subjects were divided into two groups: Group 1 (S1-S6) and Group 2 (S7, S8). Several familiarization trials were performed to ensure that the subjects understood and felt comfortable with the experimental procedures. Sufficient rest periods were given to the participants between each reaching task to avoid mental and muscle fatigue.

**Fig. 3:**
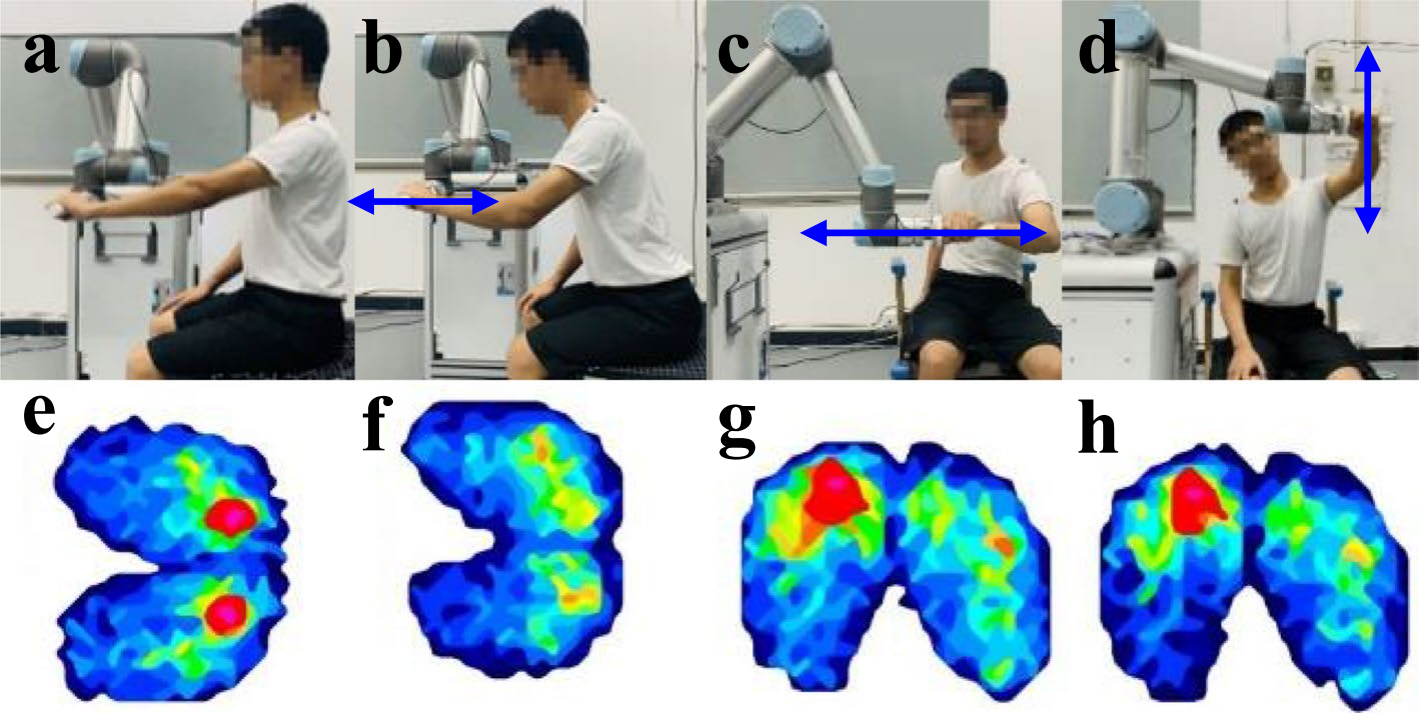
Reaching tasks and pressure maps. (**a**) Sitting straight, (**b**) back-and-forth reaching, (**c**) side-to-side reaching, (**d**) up-and-down reaching, (**e**-**h**) corresponding pressure maps

Participants sat on a chair and held onto the handle of the rehabilitation robot. The height of the chair was adjusted to keep participants’ hips and knees flexed at approximately 90°. If participants could not hold the handle, they were provided with a strap. Each subject performed three basic kinds of reaching, including (i) back-and-forth (Fig. 3b), (ii) side-to-side (Fig. 3c), and (iii) up-and-down reaching (Fig. 3d). To avoid fatigue, each participant was allowed 10 s of rest between two reaching motions and 3 min of rest after a certain type of reaching task. The total session total approximately an hour and a half on average for each participant, including resting time. Three types of compensatory patterns were commonly elicited, including an excessive trunk rotation (TR), trunk lean-forward (TLF) and shoulder-elevation (SE) [34].

#### Session 1:no feedback

No feedback was provided in this experimental trial; that is, the visual display was turned off, and the rehabilitation robot was commanded to maintain the correct direction during the execution of each motion. Each subject in Group 1 performed three kinds of reaching tasks, and each task was repeated 30 times with his/her healthy arm and affected arm. Each patient performed 180 movements in total, with 90 motions on each arm. Reaching movements performed by the participants’ healthy arm were labeled as noncompensation (NC) motions. A therapist visually monitored these moments performed by the affected arm and labeled these compensatory motions. The raw data of each subject in Group 1 were recorded as Dataset 1 for training a classifier that could be used to detect compensation from pressure distribution data. Each subject in Group 2 performed each reaching task with his/her healthy side and affected side 15 times at a self-selected speed. This process lasted about half an hour to 45 mins, including resting time. The raw data of all the participants were recorded as Dataset 2, and the classifier trained by Dataset 1 was employed to detect compensation online. The motion data of S7 and S8 were recorded by the VICON system.

#### Session 2:audiovisual feedback

**Fig. 4:**
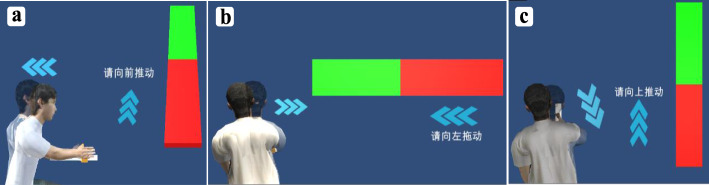
Screenshots from three reaching tasks in the virtual environment with audiovisual feedback active. (**a**) Back-and-forth reaching task, (**b**) side-to-side reaching task, and (**c**) up-and-down reaching task

Each subject performed reaching movements with his/her affected side, and therehabilitation robot was commanded to maintain the correct direction. Visual display was turned on, and audiovisual feedback was provided when the online motion recognition system detected that the participant compensated during a reaching movement, as shown in Fig. 4. Virtual scenes mainly consist of a progress bar showing the progress of subjects’ movements, an avatar instructing recommended actions to reduce compensation, and some necessary guidance in the obvious places. Subjects could see their motion from the progress bar in real time, and there was an arrow beside the progress bar indicating the direction of movement. In this study, VE resembled the traditional rehabilitation items so that participants could be immersed. The avatar did not appear on the virtual scene until compensatory motions were detected by the online compensatory pattern recognition system that we previously described. For instance, when TLF was detected during back-and-forth reaching, the avatar mimicking subjects’ movements was displayed on the screen, along with an animation of trunk lean-backward, thus introducing participants to correct compensatory motions. There was also an arrow along with the avatar, indicating the direction of decreasing compensatory motions (Fig. 4a). Simultaneously, auditory feedback was provided to inform participants of compensation and to give instructions on how to reduce compensation. Audiovisual feedback was provided similarly when patients with stroke performed side-to-side and up-and-down reaching. Each reaching task was completed 15 times in total, and the participants’ kinematics were measured by the VICON system. This process lasted about 20 mins to half an hour, including resting time.

#### Session 3:force feedback

In this experimental trial, visual display was turned off, and the rehabilitation robotprovided force feedback when a compensatory motion was detected. When participants performed reaching tasks without compensation, the rehabilitation robot was commanded to maintain the correct direction. During the familiarization trials, each participant confirmed that he could sense the change in force when compensating and ensure the assistive force was suitable for him. Each reaching task was repeated 15 times with the paretic hand, and the participant’s kinematics were recorded by the VICON system. This process lasted about 20 mins to half an hour, including resting time.

### Data preprocessing and feature extraction

The pressure maps were displayed as a color-coded real-time display in the BPMS software (Fig. 3, bottom panels). Each pressure map consists of a 32 × 32-dimensional vector, and the pressure distribution data were exported into ASCII format for processing in MATLAB. By reviewing existing research on pressure distribution mattresses [35, 36] and our previous studies [17, 18], ten features were extracted from the pressure distribution data for classification, including the average pressure sensor values (*AVE*_*SSV*_), the maximum of the pressure sensor values (*MAX*_*SV*_), the average value and standard deviation of the medial/lateral center of pressure (*AVE*_M/L-COP_ and *SD*_M/L-COP_), the average value and standard deviation of the anterior/posterior center of pressure (*AVE*_A/P-COP_ and *SD*_A/P-COP_), average value and standard deviation of the ratio of the pressure on the medial to lateral side (*AVE*_M/L-ratio_ and *SD*_M/L-ratio_), and the average value and standard deviation of the ratio of the pressure on the anterior end to the pressure on the posterior end (*AVE*_A/P-ratio_ and *SD*_A/P-ratio_).

### Classification

Based on previous studies on healthy subjects [17] and stroke patients [18], the support vector machine (SVM) classifier [37, 38] was employed to classify compensatory patterns. In this study, we trained an SVM classifier with a radial basis kernel function using LIBSVM in MATLAB [39]. Extracted features from pressure distribution data of Dataset 1 were normalized and combined in a random order before implementation of SVM classifier. The classification performance was assessed using leave-one-subject-out (LOSO) cross-validation [40]. With LOSO cross-validation, a model was trained on data from all subjects except one, who was “left out”, and the data from the one subject was used as a test dataset. The process was repeated until the data from each subject were used as a test dataset and could be used to determine the average recognition rate of the model. In the online compensation detection, an SVM classifier trained using Dataset 1 was employed to recognize compensatory patterns of subjects (S7, S8) with the aforementioned pressure features.

Confusion matrix displays information about actual and predicted classifications done by a classifier and is a convenient tool for evaluating the classification performance [41]. For binary classification, confusion matrix contains true positives (TP), true negatives (TN), false positives (FP), false negatives (FN), as shown in Table 2.

**Table 2:** Confusion matrix

		Actual Class	
		Positive	Negative
Predicted class	Positive	True positive (TP)	False positive (FP)
	Negative	False negative (FN)	True negative (TN)

Based on the confusion table, precision [42, 43] describes the accuracy of the detection and can be calculated with (1). Recall refers to how well the target objects are detected without being missed and can be calculated with (2).

1 $$Precision=\frac{TP}{TP+ FP}$$

2 $$Recall=\frac{TP}{TP+ FN}$$

The cross-validation performances of the SVM classifier were evaluated by computing overall F1-scores (harmonic mean of precision and recall) [37, 44, 45]. The F1-score can be obtained as follows:

3 $$F1=\frac{2\times Precision\times Recall}{Precision+ Recall}$$

### Statistical analysis

Differences in F1 scores were tested for statistical significance using the Friedman nonparametric tests. We conducted two-way repeated measures analyses of variance (ANOVA) (random effect: participant; fixed effect: feedback condition) to determine if compensation under the three conditions was significantly different. When the test statistic was significant, Bonferroni post hoc tests were performed to determine if differences between each of the two conditions were significant. Statistical analysis was performed using IBM SPSS statistics software (ver. 24.0, IBM Corp., Armonk, NY, USA), and a level of significance of 0.05 was selected.

## Results

### Classification performance

The offline compensatory pattern recognition performance was assessed using Dataset 1 by computing precision, recall and F1-scores, previously reported in [46]. The SVM classifier exhibited excellent performance in offline detection of compensatory patterns, with an average F1-score of 0.986 ± 0.014. The SE compensatory pattern was well detected (F1-score = 1.000), followed by TR compensation (F1-score = 0.995), NC (F1-score = 0.984) and TLF compensation (F1-score = 0.963). The Friedman nonparametric test provided statistical evidence of a significant difference in F1- scores across different compensatory patterns (*p* = 0.032). Furthermore, the performance of each class was analyzed using the SVM classifier across all subjects. The pressure distribution-based method generally classifies well (average F1-score > 0.90) for all participants. The minimum and maximum F1-scores of these four patterns among all subjects were 0.857 and 1.000, respectively.

The online classification performance in the recognition of compensatory patterns was evaluated using Dataset 2, previously reported in [46]. The SVM classifier performed well, with an average F1 score of 0.985, which indicated that the proposed method can accurately detect compensation online. The SE compensatory pattern was well detected (F1-score = 1.000), followed by TLF (F1-score = 0.992), NC (F1-score = 0.986) and TR (F1-score = 0.965). The classification accuracy was higher than 95% for both S7 and S8, which can be functionally useable for monitoring compensation in patients with stroke during reaching. Thus, based on real-time detection of compensatory patterns, different kinds of feedback, such as visual, auditory and force feedback, can be provided to participants to help them reduce compensation.

### Different feedback modalities in reducing compensation

**Fig. 5:**
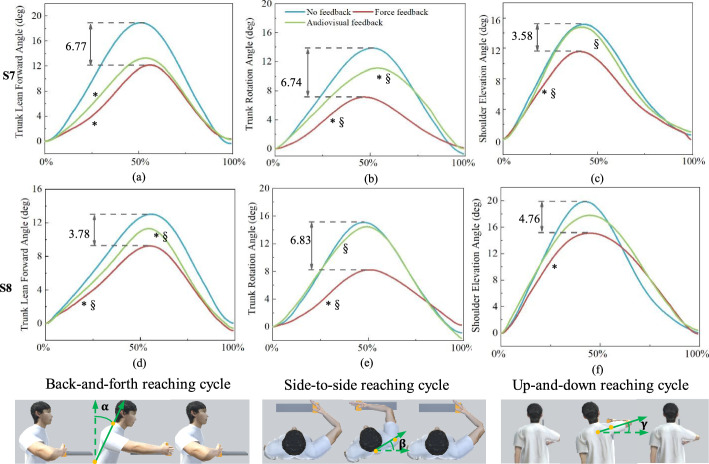
Individual results for compensation under different conditions. (**a**-**c**) represent the results of S7; (**d**-**f**) represent the results of S8. Related compensation angles are presented across the reaching cycle (back-and-forth reaching, side-to-side reaching and up-and-down reaching). The curves represent the three different conditions: no feedback (blue), audiovisual feedback (green) and force feedback (red). Note that the red curves are adopted from [46]. * indicates a significant difference (*p* < 0.05) with respect to the no-feedback condition, §indicates a significant difference (*p* < 0.05) between the audiovisual feedback and force feedback conditions

Three different reaching conditions were evaluated in the present study: no feedback, audiovisual feedback and force feedback. Fifteen motions of each reaching task per condition were used to generate mean kinematic data for each participant, which were used to compare differences among different conditions. All kinematic variables in the no-feedback condition were used as baseline comparisons. As shown in Fig. 5, compensation was measured by the angle of TLF (*α*), the angle of TR (*β*) and the angle of SE (*γ*). These three angles of S7 and S8 in the no-feedback, audiovisual feedback and force feedback conditions were analyzed. For audiovisual feedback, both S7 and S8 reduced their compensation significantly (*p* < 0.001) compared with baseline. Compared with the baseline, S7 showed greater improvement, with a reduction of 29.9, 19.8 and 2.4% in the angles of TLF, TR and SE, respectively. The maximum amount of reduction, which was 5.67°, was obtained in the back-and-forth reaching. A slight reduction of 0.36° was observed in the up-and-down reaching. For S8, the angles of TLF, TR and SE were reduced by 13.3, 4.2 and 10.3%, respectively. The maximum and minimum values of reduction were 2.05 ° and 0.63°, which were observed in up-and-down and side-to-side reaching tasks, respectively. This result indicated that effects of audiovisual feedback on different tasks differed. Specifically, significant differences (*p* < 0.001 for TLF compensation, *p* = 0.03 for TR compensation) between the audiovisual feedback and the no-feedback conditions were observed, while there was no significant difference (*p* = 0.177) in the SE compensatory pattern. Reaching with force feedback effectively reduced compensatory motions for both S7 and S8 in comparison with baseline (p < 0.001). For S7, the angles of TR, TLF and SE were reduced by 48.6, 35.7 and 23.6% in comparison with the baseline. Reduction in the side-to-side reaching task was largest, which was up to 6.77°. The minimum amount of reduction was detected in theback-and-forth reaching task, which was 3.58°. Similarly, S8 obtained a maximum reduction of 6.83° in the side-to-side reaching. When comparing the force feedback condition with the baseline, S8 reduced the angles of TR, TLF and SE by 45.3, 29.0 and 23.9%, respectively. Our observed reduction of these compensatory parameters in the audiovisual and force feedback conditions suggests that both kinds of feedback were effective in reducing compensation of patients with stroke. Surprisingly, the difference between audiovisual and force feedback was significant (*p* < 0.001), which indicated that force feedback seemed morepromising in reducing compensatory patterns in stroke survivors during reaching tasks. For S7, the angles of TR, TLF and SE were reduced by 28.8, 21.2 and 5.8% under force feedback when compared with audiovisual feedback. Reaching with force feedback led to greater decreases in compensation, especially in the side-to-side reaching task, and the maximal difference was 3.98°. For S8, compared with audiovisual feedback, which decreased the angles of TR, TLF and SE by 0.63°, 1.73° and 2.05°, respectively, reduction by using force feedback was more obvious with 6.83°, 3.78° and 4.73°, respectively. Overall, these results suggested that force feedback was a more appropriate approach to incorporate into the upper limb rehabilitation program, as it demonstrated a more efficient reduction of compensations during reaching.

## Discussion

Our first objective was to verify the feasibility of real-time monitoring compensation using a machine learning classifier from the pressure distribution data of patients with stroke. To our knowledge, this is the first time that an online pattern recognition analysis has been accomplished on compensatory motions for the population with stroke. The combination of pressure distribution data with the SVM classifier provided good classification performancein the online detection of compensations, with an average F1-score of 0.986, indicating that the pressure distribution-based system may be a viable monitoring scheme in the detection of compensatory patterns for stroke survivors during reaching tasks.

Our second objective was to investigate whether the compensatory motions of stroke survivors observed during reaching tasks can be reduced by audiovisual feedback and force feedback. Based onthe presented compensation-detection method using pressure distribution data and machine learning algorithms in real time, both audiovisual and force feedback decreased compensation exhibited by individuals with stroke in reaching; however, the effectiveness on reducing compensatory patterns varied with different individuals and reaching tasks.

Our third objective was to examine whether audiovisual feedback or force feedback was more effective in reducing compensation for patients with stroke. From the outcome measures used in this study, force feedback is a more promising approach to decreasing compensatory motions in reaching tasks. Therefore, training with force feedback is recommended for stroke patients to control motor compensation and promote motor learning during rehabilitation training.

### Online detection of compensation

Thus far, there have been no reports on the real-time monitoring of compensatory motions in patients with stroke during reaching using a machine learning model from pressure distribution data. Without inducing unnatural motions or increasing concerns related to privacy, a pressure distribution-based detection system is more portable and convenient to use in clinical and home settings than are sensor-based and camera-based systems. Our previous studies [17, 18] demonstratedthe feasibility and validity of offline recognition of compensatory patterns based on pressure distribution data and machine learning algorithms. However, offline performance is insufficient for further application in upper limb rehabilitation training, and there is usually a gap between online and offlinerecognition accuracies. An SVM classifier was trained on features from the pressure distribution data to detect three types of compensatory patterns (TLF, TF and SE) and NC in stroke patients in real time. Online recognition accuracies were higher than 95% for both S7 and S8, indicating that the method proposed in this study can be used as a real-time detection system of compensatory motions and a promising adjunct to incorporate into the upper limb rehabilitation program.

Rajiv Ranganathan et al. [13] employed a wearable sensor-based system to detect compensation in real time and obtained a classification performance with an F1-score = 0.857 for TR and an F1-score = 1.000 for SE. A camera-based system for online compensation detection has also been studied [15]; however, the classification performance was worse in stroke survivors for TR (F1-score = 0.27), TLF (F1-score = 0.17), and SE automatic detection of compensation (F1-score = 0.07). Compared to the results of previous research, our method provided more reliable accuracy of online recognition, with an average F1-score of 0.992 for TLF, 0.963 for TR, and 1.000 for SE. This evidence validated that the pressure distribution-based system can reliably detect and categorize compensatory patterns in patients with stroke during seated reaching tasks and can be used as an input for feedback systems to reduce compensation.

### Real-time reduction of compensation

Based on the motion data of stroke patients, reaching audiovisual feedback or force feedback effectively reduced compensation in comparison with the no-feedback condition (*p* < 0.001). Previous studies [19, 23, 47–50] have reported similar results for different feedback modalities on decreasing compensation in patients with stroke. Audiovisual feedbackhas been a frequent focus of research due to the popularization of VR technology. Providing auditory instructions when compensation is detected, simulating the presence of a virtual therapist, or using negative visual cues within the therapy game can improve the quality of motions so that they are more similar to therapist-supervised motions [12]. In this study, TLF and TR compensation were significantly reduced when participants were provided audiovisual feedback; however, we did not find any statistically significant difference in the SE compensatory pattern between the audiovisual feedback and the no-feedback conditions. This may indicate that the effect of audiovisual feedback is related to different levels of task difficulty. The SE compensatory pattern is mainly employed by patients with stroke in up-and-down reaching, which is also the most difficult task, as stated by participants in the experiment. Given the challenge to motor ability, the use of increased SE to aid arm and hand positioning/orientation for reaching tends to be an adaptive compensatory strategy [7]. Although audiovisual feedback was provided in real time to participants, it cannot make the up-and-down task easier. This result is consistent with the result of Norouzi-Gheidari, et al. [51], who showed that the reaching performance of patients with stroke did not differ between a physical environment and a virtual environment. As stated by Alankus, et al. [50], when audiovisual feedback is employed to remind patients of improper motor patterns, difficulty parameters need be adapted to user abilities. It is the difficulty of the motion itself that affects the improvement achieved under the audiovisual feedback condition; thus, providing audiovisual feedback is not enough in some exercises. Likewise, therapists sometimes need to correct motions, rather than only providing instruction when they supervise rehabilitation training. It may also be explained that reaching the performance of stroke patients was significantly improved (*p* < 0.001) under the force feedback condition when compared to the no-feedback or audiovisual feedback condition in this study. We noticed that neither force feedback nor audiovisual feedback was superior in reducing compensationin previous studies [23, 47]. This difference is likely the result of two kinds of force feedback. Specifically, force feedback was provided in the form of a resistive force acting in the opposite direction of motion in previous studies [23, 47], while force feedback was supplied as an assistive force acting in the same direction of motion in our study. Given that providing resistance to the participant’s limb movements makes movement tasks more difficult or challenging, providing assistive force instead of resistive force as feedback can help patients with stroke to move their affected limbs in desired patterns and reduce compensation more directly [52]. Meanwhile, the active assist strategy is the most developed control paradigm in rehabilitation robots and is widely used in robotic therapy [53]. Therefore, when compensatory patterns were detected during reaching, it was more reasonable to use rehabilitation robot to assist the impaired limb to reduce compensation and recover desired interjoint coordination.

### Limitations

There were several limitations in the current pilot study. Firstly, there were only eight patients with stroke participating in this study. Though our results suggested that force feedback showed a more promising potential than audiovisual feedback in improving reaching performance, large sample size of patients with stroke with different levels of upper limb impairment are needed to draw stronger conclusions. Secondly, the experiments were carried out in order of reaching with no feedback, reaching with audiovisual feedback and reaching with force feedback, rather than in a random order. Caution must be taken before applying our results to reducing compensation in patients with stroke and experiment with a random order is recommended. Thirdly, in this study, our classifier detect the main compensation when multiple compensation occur simultaneously. Considering that detecting compound compensation may provide more information to both the patients and therapists, we will investigate the feasibility of detecting multiple compensatory patterns. Finally, besides kinematic results, clinical outcome assessments indexes on stroke should be included in the following studies. Longitudinal studies are required to explore the long-term effects of different kinds of feedback in motor improvement and whether one kind of feedback is superior to another in the longer-term functional recovery of the upper extremities.

## Conclusion

In this study, good classification performances were obtained, with an average F1-score of 0.985 using an SVM classifier. Based on accurate online detection, rehabilitation robot and VR technologies were applied to decrease compensation in real time by providing force and audiovisual feedback to stroke patients. Effective improvements in motion patterns were observed under both the audiovisual and force feedback conditions when compared with a no feedback condition. In addition, reaching with force feedback was more promising for reducing compensation in patients with stroke.

This study is an early step in investigating feasibility of detecting compensations in patients with stroke using a machine learning algorithm from pressure distribution data in real time. Caution must be taken before applying our results to reducing compensation and further studies are required to determine the effects of choice of feedback.

## Data Availability

The datasets analyzed during the current study are available from the corresponding author on reasonable request.

## Abbreviations

BPMS: Body pressure measurement system
LOSO cross-validation: Leave-one-subject-out cross-validation
MAS: Modified Ashworth Scale
MMSE: Mini-Mental State Examination
NC: Noncompensation
SE: Shoulder elevation
SVM: Support vector machine
TCP/IP: Transmission Control Protocol/Internet Protocol
TLF: Trunk lean-forward
TR: Trunk rotation
UE-FMA: Upper extremity of the Fugl-Meyer Assessment
VAS: Visual analogue scale
VR: Virtual reality
