# Correction: Online compensation detecting for real-time reduction of compensatory motions during reaching: a pilot study with stroke survivors

**DOI:** 10.1186/s12984-025-01591-2

**Published:** 2025-05-23

**Authors:** Siqi Cai, Xuyang Wei, Enze Su, Weifeng Wu, Haiqing Zheng, Longhan Xie

**Affiliations:** 1 Shien-Ming Wu School of Intelligent Engineering, South China University of Technology, Guangzhou, 510640 China; 2 The Third Affiliated Hospital, Sun Yat-Sen University, Guangzhou, 510630 China

**Correction: Journal of NeuroEngineering and Rehabilitation (2020) 17:58** 10.1186/s12984-020-00687-1

In this article [1], the author would like to include the reference ‘Cai et al. (2020)’ in the reference list as reference 46 as shown below.

46. Cai, S., Li, G., Su, E., et al. (2020). Real-time detection of compensatory patterns in patients with stroke to reduce compensation during robotic rehabilitation therapy. IEEE journal of biomedical and health informatics, 24(9):2630–2638.

The citations of the tables 3 and 4 in the section ‘Classification performance’ should be replaced with the citation Cai et al. (2020) and both the tables have been deleted.

Classification performance

The offline compensatory pattern recognition performance was assessed using Dataset 1 by computing precision, recall and F1-scores, previously reported in [46]. The SVM classifier exhibited excellent performance in offline detection of compensatory patterns, with an average F1-score of 0.986 ± 0.014. The SE compensatory pattern was well detected (F1-score = 1.000), followed by TR compensation (F1-score = 0.995), NC (F1-score = 0.984) and TLF compensation (F1-score = 0.963).

The online classification performance in the recognition of compensatory patterns was evaluated using Dataset 2, previously reported in [46].

The caption of the Fig. 5 incorrectly appeared as.

Fig. 5 Individual results for compensation under different conditions. **a**–**c** represent the results of S7; (d–f) represent the results of S8. Related compensation angles are presented across the reaching cycle (back-and-forth reaching, side-to-side reaching and up-and-down reaching). The curves represent the three different conditions: no feedback (blue), audiovisual feedback (green) and force feedback (red). * indicates a significant difference (p < 0.05) with respect to the no-feedback condition, §indicates a significant difference (p < 0.05) between the audiovisual feedback and force feedback conditions’.

The corrected Fig. 5 caption should read as ‘Fig. 5 Individual results for compensation under different conditions. **a**–**c** represent the results of S7; **d**–**f** represent the results of S8. Related compensation angles are presented across the reaching cycle (back-and-forth reaching, side-to-side reaching and up-and-down reaching). The curves represent the three different conditions: no feedback (blue), audiovisual feedback (green) and force feedback (red). Note that the red curves are adopted from [1]. * indicates a significant difference (p < 0.05) with respect to the no-feedback condition, §indicates a significant difference (p < 0.05) between the audiovisual feedback and force feedback conditions.

The original article has been corrected.

## References

[1] Cai S, Wei X, Su E, et al. Online compensation detecting for real-time reduction of compensatory motions during reaching: a pilot study with stroke survivors. J Neuroeng Rehabil. 2020;17:58. 10.1186/s12984-020-00687-1. 32345335 10.1186/s12984-020-00687-1 PMC7189539

